# Kinetics of submaximal DFA α1 prior and post maximal efforts in trained and highly trained male and female cyclists

**DOI:** 10.1007/s00421-026-06267-w

**Published:** 2026-07-02

**Authors:** Felipe Rayo Martín, Thomas Gronwald, Peter Düking

**Affiliations:** 1 Department of Sports Science and Movement Pedagogy, Technische Universität Braunschweig, Brunswick, Germany; 2 Institute of Interdisciplinary Exercise Science and Sports Medicine, MSH Medical School Hamburg, Hamburg, Germany; 3 G-Lab, Faculty of Applied Sport Sciences and Personality, BSP Business and Law School, Berlin, Germany

**Keywords:** HRV, Autonomic nervous system, Fatigue, Readiness, Wearable

## Abstract

**Purpose:**

To investigate the kinetics of a non-linear heart rate (HR) variability parameter (DFA α1) during submaximal cycling in male and female trained and highly trained cyclists in a non-fatigued state and after repeated maximal cycling bouts.

**Methods:**

N = 47 participants (17 female) completed a cycling protocol consisting of two blocks (Block1, Block2), each including two standardised submaximal cycling bouts performed prior (PRE) and post a maximal incremental test (POST), having four time points (Block1: PRE1-POST1; Block2: PRE2-POST2). DFA α1, oxygen consumption (${\dot{\mathrm{V}}\mathrm{O}}_{{2}}$), HR, respiratory frequency (RF) and rating of perceived exertion (RPE) were assessed during the submaximal bouts, and ${\dot{\mathrm{V}}\mathrm{O}}_{{2}}$ peak and maximal power output (Wmax) during the maximal tests. A linear mixed effects model was used to analyse data.

**Results:**

Block2 showed significantly lower ${\dot{\mathrm{V}}\mathrm{O}}_{{2}}$ peak (β =  − 2.51 mL·kg⁻¹·min⁻¹) and Wmax (β =  − 24.3 W) values. DFA α1 decreased significantly from PRE1 (1.32 ± 0.19) to POST1 (1.12 ± 0.34) and remained suppressed at Block2 (1.14 ± 0.31, 1.09 ± 0.35). ${\dot{\mathrm{V}}\mathrm{O}}_{{2}}$ (+ 2.37 mL·kg⁻^1^·min⁻^1^), HR (+ 15 bpm), RF (+ 8 breaths/min) and RPE (+ 2) increased significantly in Block1 and stayed elevated in Block2. Following POST2, HR decreased (–4 bpm) and RF increased (+ 1 breaths/min) significantly. Training status significantly influenced absolute levels of DFA α1, HR, and ${\dot{\mathrm{V}}\mathrm{O}}_{{2}}$, but did not influence RPE or RF. Sex did not significantly alter kinetics of DFA α1, HR, V̇O₂, RPE and RF.

**Conclusion:**

DFA α1 during submaximal exercise reflects both acute fatigue and non-recovered state in a repeated maximal test setting and may provide complementary information on autonomic and systemic demands when interpreted alongside ${\dot{\mathrm{V}}\mathrm{O}}_{{2}}$, HR, RF and RPE.

## Introduction

Alterations in cardiorespiratory fitness and endurance performance in response to predefined training interventions exhibit considerable interindividual variability, ranging from positive to negligible or even adverse adaptations (Düking et al. 2020b). Effective load monitoring allows coaches and practitioners to identify whether an athlete is adapting appropriately to training stimuli or e.g., exhibiting signs of non-functional overreaching (Bellenger et al. 2021). A wide range of internal and external load markers, such as power output, heart rate, blood lactate, and ratings of perceived exertion have been employed to capture different aspects of the training load (Andriolo et al. 2024; Gronwald et al. 2024; Scherr et al. 2013). However, despite extensive research, there remains no single, definitive marker capable of accurately reflecting an athlete’s physiological status across all contexts and to be applicable in daily practice. Parameters are ideally obtained non-invasively, without interference with the training and recovery procedures of athletes and are assessed time efficiently. In this context, heart rate variability (HRV) derived parameters are gaining interest from the scientific community as a non-invasive method to evaluate autonomic nervous system regulation and fatigue dynamics through resting analysis (Düking et al. 2020a), as well as analysis during (Rogers and Gronwald 2022) and after exercise (Buchheit and Laursen 2013; Stanley et al. 2013), as they can be accessed by different commercially available wearable technologies (Düking et al. 2021; Rogers et al. 2025b).

Non-linear HRV methods, particularly Detrended Fluctuation Analysis (DFA), offer capturing the complex, fractal nature of heart rate dynamics. The short-term scaling exponent alpha1 (DFA α1) (Peng et al. 1995) quantifies correlations within inter-beat interval time series and reflects the degree of self-similarity in cardiac autonomic regulation. Previous studies have demonstrated that DFA α1 decreases progressively with increasing exercise intensity, transitioning from well-correlated fractal patterns toward uncorrelated or random dynamics as physiological stress accumulates (Rogers and Gronwald 2022).

Next to the demarcation of exercise intensity thresholds (Kanniainen et al. 2023), DFA α1 has been shown to be useful as an additional marker of acute fatigue in terms of systemic perturbation patterns in HR time series (Rogers et al. 2021c; Van Hooren et al. 2023b) or as a measure of fatigue resistance in studies with prolonged exercise (Gronwald et al. 2024). These properties position DFA α1 as a potentially valuable marker for assessing internal load, fatigue, and “readiness to train” in both laboratory and field environments (Gronwald et al. 2025; Rogers et al. 2021c; Rogers and Gronwald 2022; Schaffarczyk et al. 2022a). Despite these promising applications, more research is needed around areas of DFA α1 during exercise as most of the investigations have focused on incremental exercise protocols or relatively small and homogeneous cohorts, which may limit the generalizability of the findings (Tanner et al. 2024). Moreover, the kinetics of DFA α1 during submaximal cycling performed before and after repeated maximal exercise bouts, representing a condition of acute fatigue accumulation, have not yet been fully characterized (Ajayi et al. 2025; Gronwald et al. 2019; Van Hooren et al. 2023a). A recent study (Van Hooren et al. 2023a) investigated DFA α1 measured during a standardized low-intensity exercise bout performed before and after an incremental treadmill test. Their findings suggested that DFA α1 may be useful to monitor physiological status during both non-fatigued and fatigued conditions. That investigation primarily focused on reliability aspects in a relatively small cohort (n = 10) within a running specific protocol. While this work has increased our understanding of DFA α1 during submaximal runs, additional work is needed to examine DFA α1 kinetics in larger samples and across repeated maximal exercise bouts designed to induce acute fatigue. Additionally, whether DFA α1 kinetics respond similarly across sexes (Mendonca et al. 2010; Sheoran et al. 2024) and performance levels remains unclear (Rogers and Gronwald 2022), as previous studies have largerly focused on incremental exercise protocolos and have not specifically adressed fatigue-induced alterations during subamximal exercise.

Therefore, the aims of the present study are (i) to assess the exercise induced potential alterations of DFA α1 and other internal load parameters (HR, oxygen consumption [${\dot{\mathrm{V}}\mathrm{O}}_{{2}}$], rating of perceived exertion [RPE], respiratory frequency [RF]) during submaximal cycling prior to and following repeated maximal exercise bouts in a larger sample of participants, as well as (ii) to investigate potential differences between trained and highly trained individuals, and between male and female athletes.

We hypothesize that repeated maximal exercise bouts would induce significant alterations in autonomic regulation and internal load responses during subsequent submaximal cycling. Specifically, we expected DFA α1 values to be reduced and other internal load parameters (HR, ${\dot{\mathrm{V}}\mathrm{O}}_{{2}}$, RPE, and RF) to be elevated following repeated maximal exercise compared to pre-exercise conditions. In addition, we hypothesize that the magnitude of these responses would differ between trained and highly trained individuals, with highly trained athletes exhibiting attenuated alterations. Finally, we expect no potential sex-specific differences in the response pattern.

## Methods and materials

### Participants

N = 52 healthy participants (32 male, 20 female) participated in the study. Participants with cardiovascular, neurological, orthopaedic, metabolic, or respiratory disorders, acute illness, or medication affecting exercise capacity were excluded. Female participants were required to have a regular menstrual cycle and no menstrual distress in the preceding six months. The study adhered to the Declaration of Helsinki, and all participants provided written informed consent. The ethics committee of TU Braunschweig approved the protocol (reference no. FV-2025–06).

Participants with data files with artifacts over 5% were identified and not used in any further analysis. This threshold has previously been shown to have minimal influence on DFA α1 during exercise (Rogers et al. 2021a, b). Five participants were excluded due to malfunctioning of the recording app. Thus, a total number of 47 participants were included in the final analysis and participants characteristics are summarized in Table 1. Participants were classified as highly trained (n = 25) or trained (n = 22) cyclists according to a recently published classification framework (McKay et al. 2022).

**Table 1:** Descriptive data of each participant and group divisions

Group	n =	Mean ± SD				
		${\dot{\mathrm{V}}\mathrm{O}}_{{2}}$ peak (mL·min⁻^1^·kg⁻^1^)	Age (years)	Height (cm)	Body mass (kg)	BMI (kg/m^2^)
Male	30	57.99 ± 8.93	27 ± 5	180.4 ± 6.8	76.3 ± 9.0	23.4 ± 2.3
Highly trained	16	64.72 ± 4.70	26 ± 3	181.6 ± 6.1	73.7 ± 7.7	22.3 ± 1.6
Trained	14	50.29 ± 5.72	29 ± 6	179.0 ± 7.5	79.2 ± 9.8	24.7 ± 2.4
Female	17	46.48 ± 7.99	27 ± 3	167.9 ± 4.5	66.4 ± 7.0	23.5 ± 2.1
Highly trained	9	52.58 ± 3.89	27 ± 2	168.2 ± 3.3	64.6 ± 4.9	22.8 ± 1.3
Trained	8	39.63 ± 5.20	26 ± 3	167.6 ± 5.9	68.5 ± 8.6	24.4 ± 2.7
Total	47	53.83 ± 10.18	27 ± 4	175.9 ± 8.6	72.7 ± 9.5	23.5 ± 2.2

*n* sample size per group, $\dot{V}O_{2}$ *peak* peak oxygen consumption, *BMI* body mass index

## Study design

### Testing procedures

As previously employed (Zadow et al. 2016, 2018), all participants performed on an electronically braked cycle ergometer (Wahoo KICKR, Wahoo Fitness, Atlanta, GA, USA). The ergometer was calibrated prior to each test using the manufacturer’s proprietary software. Participants were permitted to use their own bicycles, or were provided with a standardized road bicycle, with individual adjustments made to saddle and handlebar positions.

The experimental design is conceptually comparable to previously described ramp-based incremental testing protocols (Van Hooren et al. 2023a), which were conducted in treadmill running. Our protocol was divided in two identical blocks (Block1, Block2). The exercise protocol began in Block1 with an initial 5-min submaximal monitoring period (PRE1), consisting of 5 min at a constant workload of 100 W for male participants (mean relative intensity 1.3 ± 0.2 W·kg⁻^1^) and 70 W for female participants (mean relative intensity: 1.1 ± 0.1 W·kg⁻^1^) to assess unfatigued DFA α1 and the other parameter values of each participant. Participants were instructed to keep a pedalling frequency between 70 and 100 revolutions per minute and to remain seated throughout all exercise trials. Thereafter, participants performed the first incremental ramp test (Ramp1), consisting of a continuous increase in workload of + 15 W every 30 s until volitional exhaustion. Maximal exertion and attainment of maximal oxygen uptake (${\dot{\mathrm{V}}\mathrm{O}}_{{2}}$peak) were confirmed when all three of the following criteria were met: (i) a plateau in ${\dot{\mathrm{V}}\mathrm{O}}_{{2}}$ despite increased workload and ventilation; (ii) respiratory exchange ratio (RER) ≥ 1.10; and (iii) RPE ≥ 18.

Following Ramp1, participants performed a 3-min period of passive, seated recovery period on the bike, followed by a second monitoring submaximal monitoring period (POST1) to assess parameter values.

**Fig. 1:**
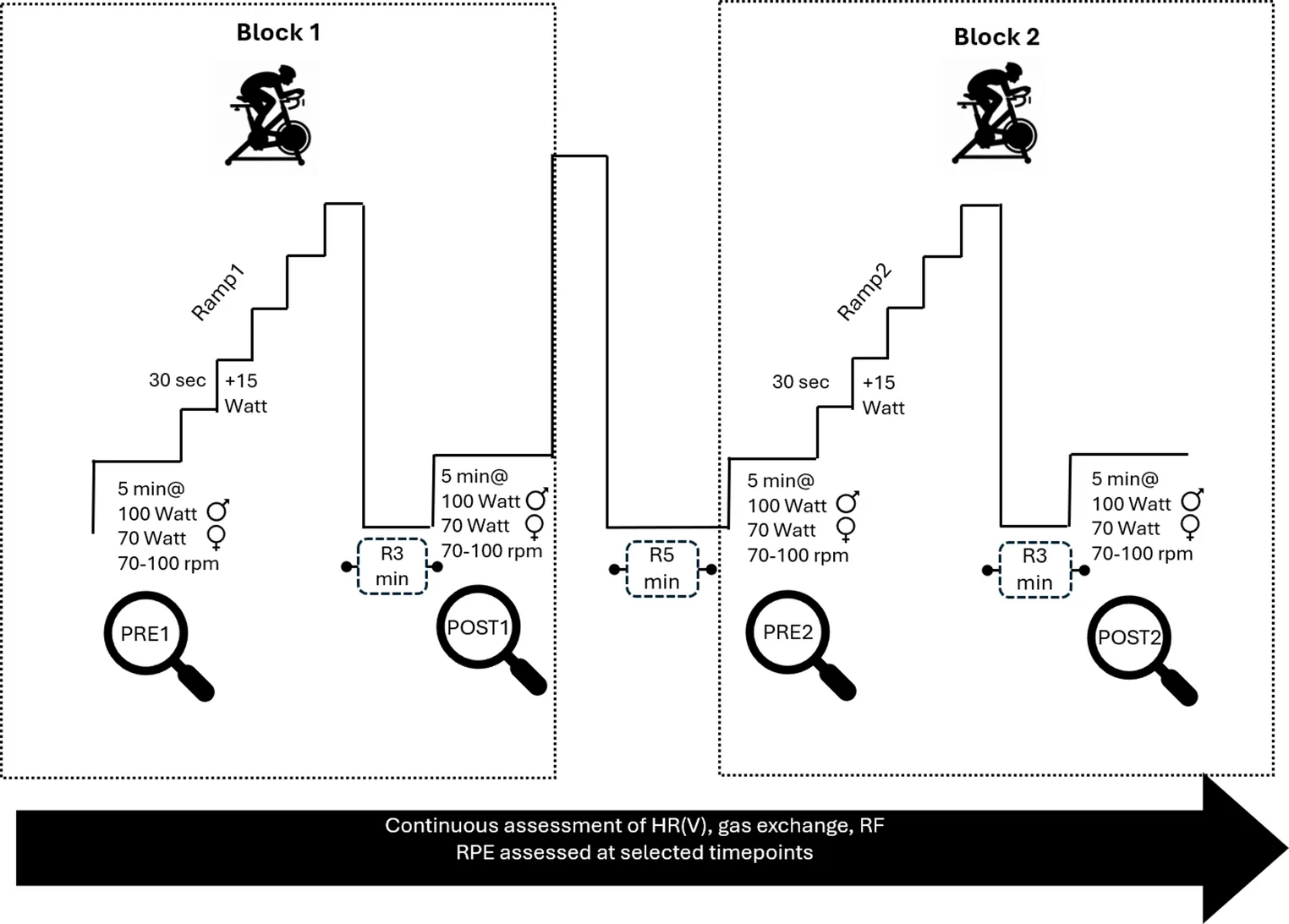
Schematic representation of experimental procedures. Gas exchange, HR(V): heart rate (variability), RPE: Rating of perceived exertion, RF: respiratory frequency, R3min: recovery 3 min. R5min: recovery 5 min

Experimental procedures are schematically depicted in Fig. 1. The herein presented data analysis was part of a larger investigation around performance analysis of trained and highly trained cyclists.

To induce additional fatigue and to confirm ${\dot{\mathrm{V}}\mathrm{O}}_{{2}}$peak, participants completed between Block1 and Block2 a verification trial at the workload corresponding to the maximal power output (Wmax) at the final fully completed stage of Ramp1, performed to volitional exhaustion. This procedure followed established recommendations for ${\dot{\mathrm{V}}\mathrm{O}}_{{2}}$peak verification (Poole and Jones 2017). If ${\dot{\mathrm{V}}\mathrm{O}}_{{2}}$peak obtained during the verification trial exceeded that measured during Ramp1 and these values were above the 3% measurement error of the gas analyzer (Van Hooren et al. 2024), the higher value was adopted. However, none of the athletes showed an at least 3% higher ${\dot{\mathrm{V}}\mathrm{O}}_{{2}}$peak in the verification trial compared to the value obtained during Ramp1.

Following the verification trial, participants performed a 3-min period of passive, seated recovery period on the bike, followed by Block2 with a third monitoring submaximal monitoring period (PRE2) to assess parameter values.

Afterwards, participants performed a second incremental ramp test (Ramp2) identical to Ramp1. A final 3-min passive recovery was provided before the protocol concluded with a fourth 5-min submaximal monitoring period (POST2). All testing sessions were performed under standardized laboratory conditions with ambient temperature maintained between 19 and 23 °C and relative humidity between 40 and 60%. Participants were instructed to refrain from engaging in high-intensity exercise for at least 48 h prior to testing to ensure sufficient recovery. To standardize pre-exercise nutritional status, participants were required to consume their final meal at least 2 h before testing and a carbohydrate-rich meal was recommended to optimize energy availability. No fluid or nutritional intake was permitted during testing. Given that all participants were experienced cyclists and trained regularly, a familiarization session was not deemed necessary. Additionally, a fan was provided during all tests and positioned diagonally at approximately one meter from the rider to facilitate cooling.

### Assessment of anthropometrics and gas exchange parameters

Upon arrival to the testing facility, anthropometric characteristics including body mass, body height and body mass index were assessed. Body mass was measured using a bioelectrical impedance device (Tanita BC-601, Tokyo, Japan), while body height was determined using a standard measuring tape. Pulmonary gas exchange variables were measured breath-by-breath using a calibrated gas analyzer (Metalyzer 3B R2, Cortex Biophysik, Leipzig, Germany). The analyzer was calibrated before each test with a certified reference gas (15.8% O₂, 5% CO₂ in N₂; Praxair, Düsseldorf, Germany) to reflect physiological gas fractions. Flow volume accuracy was verified with a precision 3-L calibration syringe. The herein used breathing gas analyzer has an error of 3% when assessing V̇O₂peak values as evaluated on a metabolic simulator in “CPX mode” (Van Hooren et al. 2024).

During the test, RPE (scale 6–20) (Scherr et al. 2013) was recorded. Athletes were instructed to self-assess their perceived exertion and to provide immediate and precise responses to minimize artifacts associated with verbal reporting.

### Assessment of DFA α1

DFA α1 and HR was continuously assessed using a chest strap HR monitor (Polar H10, Polar Electro Oy, Kempele, Finland) providing RR interval data suitable for HRV analysis (Schaffarczyk et al. 2022b). Consistent with its application in recent previous studies (Rogers et al. 2025a; Van Hooren et al. 2023a, b), signals were acquired via Bluetooth through the open-source Fatmaxxer application (version available at: https://github.com/IanPeake/FatMaxxer), operated on a Samsung Galaxy S10 + smartphone (Samsung Electronics, Suwon, South Korea). Signal quality was assessed using the Fatmaxxer application, which displays the R-wave peak voltage. Electrode belt placement was adjusted when necessary to ensure stable signal detection.

The Fatmaxxer application provides real-time processing of R-R intervals, including artifact detection, percentage artifact quantification, and DFA α1 calculations. The validity of this open-source App has been proven recently, showing the DFA α1 responses closely aligned with those obtained from the established standard Kubios HRV software (Rogers et al. 2025b). Although the application implements the Kubios “smoothness priors” detrending method (Tarvainen et al. 2002) raw R-R interval data were ultimately reprocessed using Kubios HRV Scientific Lite Software Version 4.1.2. Preprocessing settings for Kubios HRV were set to the default values, including the R-R detrending method, which was kept at “smoothness priors” (Lambda = 500). The R-R interval series were then corrected using the Kubios HRV “threshold correction” method set at medium. Previous evidence indicates that artefact correction can introduce bias in DFA α1 estimates, with the magnitude and direction of bias depending on the underlying DFA α1 level. Artefact correction has been shown to falsely increase DFA α1 values, particularly within the low, uncorrelated and anticorrelated range. In contrast, values close to 1.0 appear to be minimally affected, whereas values near 0.5 may be moderately affected (Rogers et al. 2021b). Given that DFA α1 in the present study was assessed during standardized submaximal exercise bouts and predominantly remained within the correlated range, the potential impact of artefact-related bias on the present results is considered limited. For DFA α1 calculation window size was set to 4 ≤ n ≤ 16 beats in the software preferences. Data were additionally scanned visually for artefacts (e.g., spikes in R-R interval series), marked as “noise” and removed manually if necessary. In such cases, the start and duration of affected segments were identified based on the time axis, and the corresponding RR intervals were subsequently removed using Microsoft Excel. Datasets with > 5% artefacts were excluded from analysis. DFA α1 was calculated from a single 2-min R-R interval segment extracted from minute 4 to 5 of the submaximal 5 min bouts (PRE1 and 2, POST1 and 2).

### Statistics

Since each participant was measured at four time points (PRE1, PRE2, POST1, POST2), to examine the effects of exercise induced acute (after the first exertion from PRE1 to POST1) and accumulated fatigue (exertion in PRE2 and POST2) on variables (DFA α1, HR, V̇O₂, RF, RPE) a linear mixed-effects model (LMM) was fitted using GAMLj3 (Gallucci 2023) in Jamovi with Restricted Maximum Likelihood (REML). Before analysis, we defined DFA α1 as the primary outcome and all other outcomes were treated as secondary (exploratory). This distinction follows current guidance for transparent reporting of multilevel and mixed-model analyses (Monsalves et al. 2020). The LMM for the primary outcome was considered confirmatory, whereas results for secondary outcomes are interpreted cautiously and their p-values are presented descriptively without formal adjustment for multiple testing. The fixed effects were divided into within-subject and between-subject factors. The within-subject structure was represented by two factors: Block (Block1 vs. Block2) and Timepoint (PRE vs. POST within each block). Between-subject factors were Sex (male vs. female) and Performance level (trained vs. highly trained). We then fitted a LMM of the form:

$$\begin{aligned}Y_{ij \,} &= \beta_{0} + \, \beta_{1} Block_{ij} + \, \beta_{2} Time_{ij} + \, \beta_{3} Sex_{i} \\ & \quad + \, \beta_{4} Performance_{i} + \, all \, interactions \, + \, u_{0i} + \, \varepsilon_{ij} \end{aligned}$$

where *Y*_*ij*_ is the outcome for person *i* at measurement *j*, *u*_*0i*_ is a random intercept for participant (capturing between-person differences), and *ε*_*ij*_ is the residual error term (Lu et al. 2024). Omnibus tests showed overall contribution of each fixed effect and statistical significance was set at p < 0.05, evaluated using Type III F-tests with Kenward-Roger approximations denominator degrees of freedom (Kenward and Roger 1997). In our model, the main effect of Block tested whether overall levels differ between Block1 and Block2, averaged across Timepoint and groups (Sex, Performance level). The main effect of Timepoint tests whether there is an overall PRE-to-POST change across both blocks. The Block*Timepoint interaction addresses whether the magnitude of the PRE-POST change differs between the two blocks. Interactions of Block and/or Timepoint with Sex and Performance level test whether these temporal patterns differ between groups.

To quantify model fit we report marginal and conditional coefficient of determination (R^2^) according to Nakagawa et al. (2017) where the marginal R^2^ reflects variance explained by the fixed effects and the conditional R^2^ reflects variance explained by both fixed and random effects (Nakagawa et al. 2017). This allowed us to see how much of the variability in the outcome is due to the experimental factors (Block, Timepoint, Sex, Performance level) and how much is due to individual differences. To verify that the repeated testing protocol induced measurable fatigue, we also analysed maximal oxygen uptake (${\dot{\mathrm{V}}\mathrm{O}}_{{2}}$peak) and maximal power output (Wmax) with LMM including Block (1 vs. 2), Sex and Performance Level (trained vs. highly trained) as fixed effects and a random intercept for participant.

Model assumptions (normality and homoscedasticity) were assessed using Q-Q plots and residual vs. fitted plots, respectively, following standard recommendations (Zuur et al. 2009). No major violations were observed. Given evidence that LMM are robust to moderate violations of the normality assumption and mild heteroscedasticity, especially in balanced designs (Knief and Forstmeier 2021) the model assumptions were considered adequately met if residuals showed normal distribution with only minor deviations in the extremes of the predicted range. Otherwise, we repeated the analysis on log-transformed data as sensitivity analyses. If these checks achieved the same qualitative pattern of results, we reported the estimates from the original scale for ease of interpretation.

To report the means from the predictions of the statistical model, we extracted the values using Estimated Marginal Means (EMMs) based on the emmeans R package (Lenth and Piaskowski 2025). Pairwise Post-Hoc comparisons with Tukey’s adjustment were performed, where a significant overall difference was found, to identify exactly which specific pairs of groups are significantly different from each other. All statistical analyses were conducted in Jamovi (version 2.7), RStudio (version 2025.05.1 + 513) and Microsoft Excel (Microsoft Corp., Redmond, WA, USA) on Mac OS (Version 15.6.1).

## Results

Overall ${\dot{\mathrm{V}}\mathrm{O}}_{{2}}$ peak decreased significantly from Block1 to Block2 (β =  − 2.51 mL/kg/min, 95% CI − 2.74 to − 2.02, p < 0.001), corresponding to a 4.7% reduction across groups. Wmax also decreased significantly from Block1 to Block2 (β =  − 24.3 W, 95% CI − 27.5 to − 21.6, p < 0.001), corresponding to a 6.7% reduction (see Table 2). Descriptive data for the internal load parameters measured during the standardized submaximal exercise bouts are also presented in Table 3.

**Table 2:** Maximal performance outcomes across the Blocks reported as mean ± SD for the total sample as well as divided by sex and performance level

Variable	Block1 (mean ± SD)	Block2 (mean ± SD)	Δ Block2–Block1
${\dot{\mathrm{V}}\mathrm{O}}_{{2}}$ peak (mL·kg⁻^1^·min⁻^1^)	53.83 ± 10.18	51.32 ± 9.66	− 2.51 (− 4.7%)
Highly trained	60.35 ± 7.29	57.69 ± 5.90	− 2.66 (− 4.4%)
Trained	46.41 ± 7.45	44.09 ± 7.69	− 2.32 (− 5.0%)
Male	57.99 ± 8.86	55.15 ± 7.87	− 2.84 (− 4.9%)
Highly trained	64.72 ± 4.63	61.22 ± 3.34	− 3.50 (− 5.4%)
Trained	50.29 ± 5.62	48.22 ± 5.39	− 2.07 (− 4.1%)
Female	46.48 ± 7.87	44.56 ± 8.70	− 1.92 (− 4.1%)
Highly trained	52.58 ± 3.78	51.41 ± 3.81	− 1.17 (− 2.2%)
Trained	39.63 ± 5.02	36.86 ± 5.47	− 2.77 (− 7.0%)
Wmax (W)	360.1 ± 77.8	336.2 ± 76.0	− 23.9 (− 6.7%)
Highly trained	394.0 ± 66.2	372.4 ± 58.4	− 21.6 (− 5.5%)
Trained	321.6 ± 73.0	295.0 ± 73.6	− 26.6 (− 8.3%)
Male	396.5 ± 61.6	369.0 ± 64.6	− 27.5 (− 7.0%)
Highly trained	430.9 ± 47.6	402.8 ± 45.2	− 28.1 (− 6.5%)
Trained	357.1 ± 62.2	330.4 ± 62.7	− 26.8 (− 7.5%)
Female	295.9 ± 52.4	278.2 ± 58.8	− 17.6 (− 6.0%)
Highly trained	328.3 ± 36.6	318.3 ± 35.3	− 10.0 (− 3.1%)
Trained	259.4 ± 43.1	233.1 ± 45.7	− 26.3 (− 10.1%)

The absolute and relative change from Block1 to Block2 is also shown

*SD* standard deviation, $\dot{V}O_{2}$*peak* maximal oxygen uptake, *Wmax* maximal power output. *Δ* Delta Block2 – Block1

**Table 3:** Submaximal responses before and after maximal efforts across blocks

Variable	n	Block1		Block2	
		PRE1	POST1	PRE2	POST2
*DFAa1*	47	1.32 ± 0.19	1.12 ± 0.34	1.14 ± 0.31	1.09 ± 0.35
Highly trained	25	1.36 ± 0.17	1.17 ± 0.33	1.22 ± 0.30	1.19 ± 0.28
Trained	22	1.28 ± 0.21	1.07 ± 0.35	1.05 ± 0.31	0.99 ± 0.40
Male	30	1.34 ± 0.16	1.12 ± 0.33	1.15 ± 0.32	1.14 ± 0.36
Highly trained	16	1.39 ± 0.12	1.13 ± 0.38	1.19 ± 0.37	1.22 ± 0.34
Trained	14	1.28 ± 0.19	1.12 ± 0.28	1.10 ± 0.27	1.05 ± 0.36
Female	17	1.28 ± 0.24	1.12 ± 0.36	1.12 ± 0.31	1.01 ± 0.34
Highly trained	9	1.30 ± 0.24	1.25 ± 0.21	1.27 ± 0.11	1.13 ± 0.09
Trained	8	1.27 ± 0.25	0.99 ± 0.45	0.96 ± 0.38	0.88 ± 0.46
$\dot{V}O_{2}$* (mL·kg⁻*^*1*^*·min⁻*^*1*^*)*	47	21.90 ± 3.12	24.41 ± 3.34	23.03 ± 3.17	23.29 ± 3.05
Highly trained	25	23.63 ± 2.50	25.78 ± 2.80	24.41 ± 2.54	24.70 ± 2.30
Trained	22	19.94 ± 2.56	22.86 ± 3.27	21.48 ± 3.15	21.69 ± 3.04
Male	30	22.90 ± 2.60	25.83 ± 2.67	24.37 ± 2.34	24.49 ± 2.50
Highly trained	16	24.39 ± 2.16	26.92 ± 2.41	25.37 ± 2.00	25.64 ± 1.75
Trained	14	21.20 ± 1.97	24.58 ± 2.46	23.24 ± 2.24	23.18 ± 2.63
Female	17	20.14 ± 3.23	21.91 ± 2.95	20.67 ± 3.12	21.18 ± 2.82
Highly trained	9	22.27 ± 2.60	23.75 ± 2.31	22.69 ± 2.57	23.04 ± 2.30
Trained	8	17.75 ± 1.94	19.84 ± 2.14	18.40 ± 1.87	19.09 ± 1.64
*HR (bpm)*	47	117 ± 12	133 ± 14	135 ± 13	130 ± 13
Highly trained	25	114 ± 11	130 ± 14	130 ± 13	124 ± 11
Trained	22	120 ± 12	137 ± 13	139 ± 13	136 ± 14
Male	30	115 ± 10	133 ± 13	135 ± 13	129 ± 13
Highly trained	16	115 ± 12	134 ± 14	134 ± 13	127 ± 11
Trained	14	115 ± 9	133 ± 13	136 ± 13	132 ± 14
Female	17	120 ± 13	132 ± 15	134 ± 15	131 ± 15
Highly trained	9	113 ± 10	122 ± 12	124 ± 10	120 ± 8
Trained	8	129 ± 11	143 ± 11	146 ± 9	144 ± 9
*RF (breaths/min)*	47	22 ± 4	30 ± 6	30 ± 6	31 ± 5
Highly trained	25	22 ± 4	30 ± 7	30 ± 6	30 ± 5
Trained	22	22 ± 3	30 ± 5	30 ± 4	32 ± 5
Male	30	21 ± 4	30 ± 6	29 ± 5	30 ± 5
Highly trained	16	21 ± 4	31 ± 7	30 ± 6	30 ± 5
Trained	14	21 ± 3	29 ± 4	28 ± 4	31 ± 5
Female	17	23 ± 3	29 ± 6	31 ± 6	32 ± 6
Highly trained	9	23 ± 3	28 ± 7	29 ± 7	29 ± 6
Trained	8	24 ± 3	31 ± 5	32 ± 4	34 ± 5
*RPE (6–20)*	47	7 ± 1	10 ± 2	10 ± 3	10 ± 3
Highly trained	25	7 ± 1	10 ± 2	10 ± 3	10 ± 3
Trained	22	7 ± 1	10 ± 2	10 ± 2	10 ± 3
Male	30	7 ± 1	10 ± 2	10 ± 3	10 ± 3
Highly trained	16	7 ± 1	10 ± 3	10 ± 3	10 ± 3
Trained	14	7 ± 1	9 ± 2	10 ± 2	9 ± 2
Female	17	8 ± 1	10 ± 2	11 ± 2	10 ± 3
Highly trained	9	7 ± 2	9 ± 2	10 ± 2	10 ± 3
Trained	8	8 ± 1	11 ± 2	11 ± 2	11 ± 3

Group mean ± SD measured during submaximal cycling bouts performed before (PRE) and after (POST) the time-to-exhaustion test in Block1 (PRE1, POST1) and Block2 (PRE2, POST2), for the total sample and divided by sex and performance level

*SD* standard deviation, *DFA α1* short-term scaling exponent alpha1, $\dot{V}O_{2}$ oxygen consumption, *HR* heart rate, *RF* respiratory frequency, *RPE* rating of perceived exertion, *n* sample size per group.

**Fig. 2:**
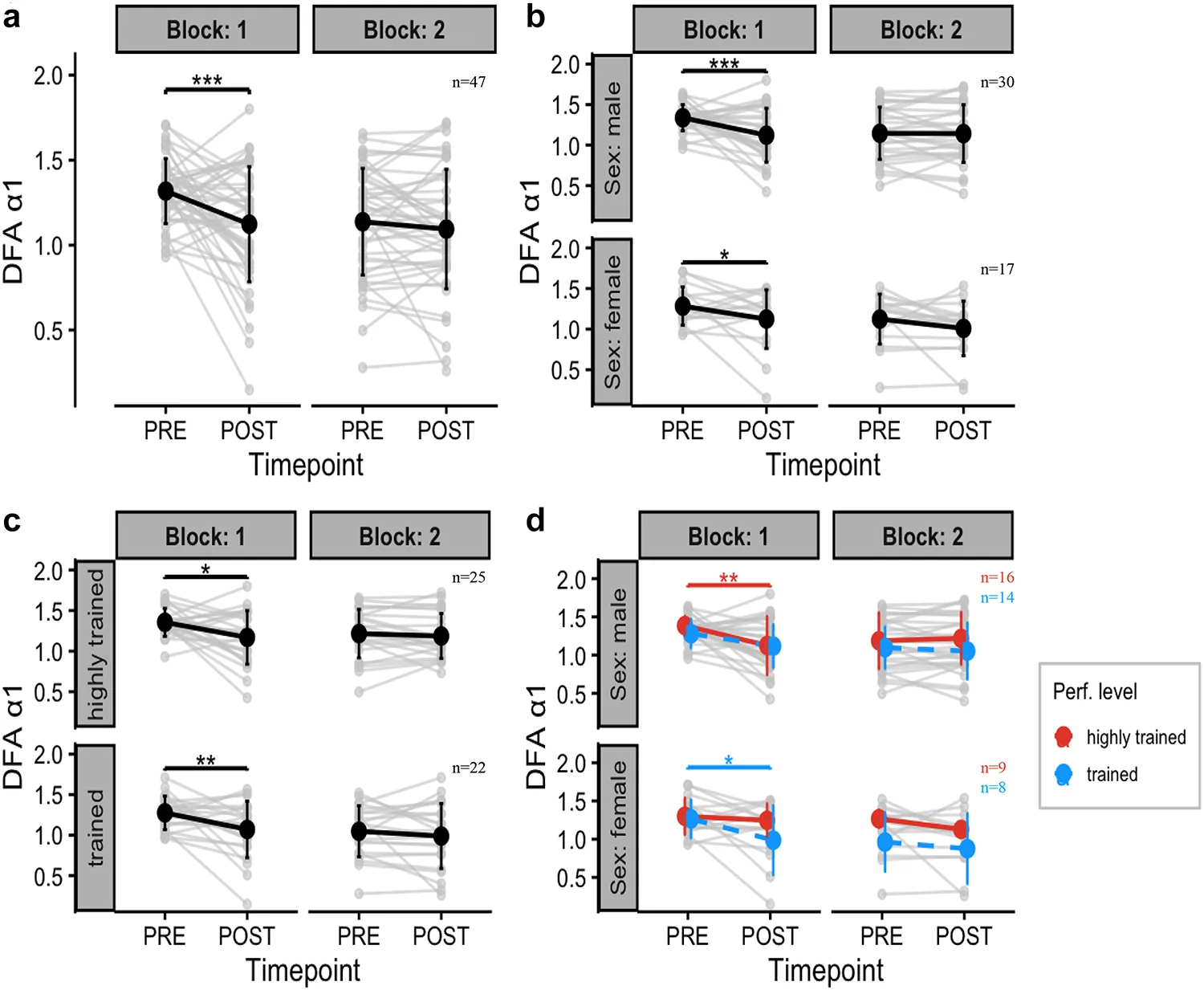
Kinetics of the short-term scaling exponent of detrended fluctuation analysis (DFA α1) during submaximal cycling bouts performed before (PRE) and after (POST) the time-to-exhaustion effort in Block1 and Block2. **a** All participants pooled. **b** divided by sex (male, female). **c** divided by performance level (highly trained, trained). **d** Sex-specific responses shown separately for performance level (perf. Level) (red = highly trained; blue = trained). Grey lines represent individual participant responses; group means are shown as points with error bars (± 1 SD). Asterisks indicate significant PRE—POST differences within the corresponding block (* p < 0.05; ** p < 0.01; *** p < 0.001)

The primary outcome DFA α1 showed to be significantly affected by Performance level (F₁, ₄₃ = 5.15, p = 0.028). Compared to highly trained participants, the trained group showed lower DFA α1 values overall (β =  − 0.15, 95% CI − 0.29 to − 0.02). DFA α1 also differed between Blocks (F₁, ₁₂₉ = 10.28, p = 0.002) and Timepoints (F₁, ₁₂₉ = 11.97, p < 0.001). Values were lower in Block2 than Block1 (β =  − 0.12, 95% CI − 0.19 to − 0.04) and Timepoint showed an overall effect (β =  − 0.12, 95% CI − 0.20 to − 0.05) (see Table 4). Post-hoc comparisons of the Block*Timepoint interaction revealed that DFA α1 was highest at the beginning of Block1 (PRE1) and significantly lower at POST1 (p < 0.001) (see Fig. 2). Differences between the three later time points were small and not statistically significant. No interactions between sexes or performance levels reached significance.

**Table 4:** Linear mixed-effects model results for submaximal and maximal outcomes

Outcome variable	Marginal R^2^	Conditional R^2^	Performance level β (95% CI); p	Block β (95% CI); p	Timepoint β (95% CI); p	Sex β (95% CI); p	Block1 Δ(POST–PRE) β (95% CI); p	Block2 Δ(POST–PRE) β (95% CI); p
DFA α1	0.15	0.48	− 0.15 (− 0.29, − 0.02); p = 0.028	− 0.12 (− 0.19, − 0.04); p = 0.002	− 0.12 (− 0.20, − 0.05); p < 0.001	− 0.06 (− 0.19, 0.08); p = 0.417	− 0.19 (− 0.32, − 0.06); p < 0.001	− 0.06 (− 0.19, 0.07); p = 0.224
${\dot{\mathrm{V}}\mathrm{O}}_{{2}}$ (mL·kg⁻^1^·min⁻^1^)	0.56	0.93	− 3.35 (− 4.60, − 2.10); p < 0.001	− 0.01 (− 0.26, 0.25); p = 0.958	1.34 (1.09, 1.60); p < 0.001	− 3.46 (− 4.71, − 2.21); p < 0.001	2.37 (1.89, 2.85); p < 0.001	0.32 (− 0.16, 0.79); p = 0.089
HR (bpm)	0.41	0.89	11 (5, 18); p = 0.001	7 (6, 9); p < 0.001	5 (4, 7); p < 0.001	2 (− 5, 8); p = 0.595	15 (12, 18); p < 0.001	− 4 (− 7, − 2); p < 0.001
RF (breaths/min)	0.36	0.83	1 (− 2, 4); p = 0.416	4 (4, 5); p < 0.001	4 (4, 5); p < 0.001	1 (− 1, 4); p = 0.371	8 (6, 9); p < 0.001	1 (0, 3); p = 0.045
RPE (6–20)	0.22	0.72	0 (− 1, 2); p = 0.450	1 (1, 2); p < 0.001	1 (1, 2); p < 0.001	1 (0, 2); p = 0.244	2 (2, 3); p < 0.001	0 (− 1, 1); p = 0.371
${\dot{\mathrm{V}}\mathrm{O}}_{{2}}$peak (mL·kg⁻^1^·min⁻^1^)	0.77	0.99	− 13.74 (− 16.55, − 10.92); p < 0.001	− 2.38 (− 2.74, − 2.02); p < 0.001		− 10.99 (− 13.81, − 8.18); p < 0.001		
Wmax (W)	0.60	0.99	− 74.1 (− 104.7, − 45.5); p < 0.001	− 22.8 (− 25.3, − 20.3); p < 0.001		− 95.5 (− 125.1, − 65.9); p < 0.001		

Marginal and conditional R^2^ values and fixed-effect estimates (β, 95% CI, p) for performance level, block, timepoint (PRE vs POST), and sex for all the variables. Estimated within-block PRE–POST changes (ΔPOST–PRE) are reported separately for Block1 and Block2

*R*^*2*^ coefficient of determination, *CI* confident interval, *β* effect´s regression coefficients, Submaximal outcomes: *DFA α1* short-term scaling exponent alpha1, $\dot{V}O_{{2}}$ oxygen consumption, *HR* heart rate, *RF* respiratory frequency, *RPE* rating of perceived exertion, Maximal outcomes: $\dot{V}O_{{2}}$*peak* peak oxygen consumption, *Wmax* maximal power output

**Fig. 3:**
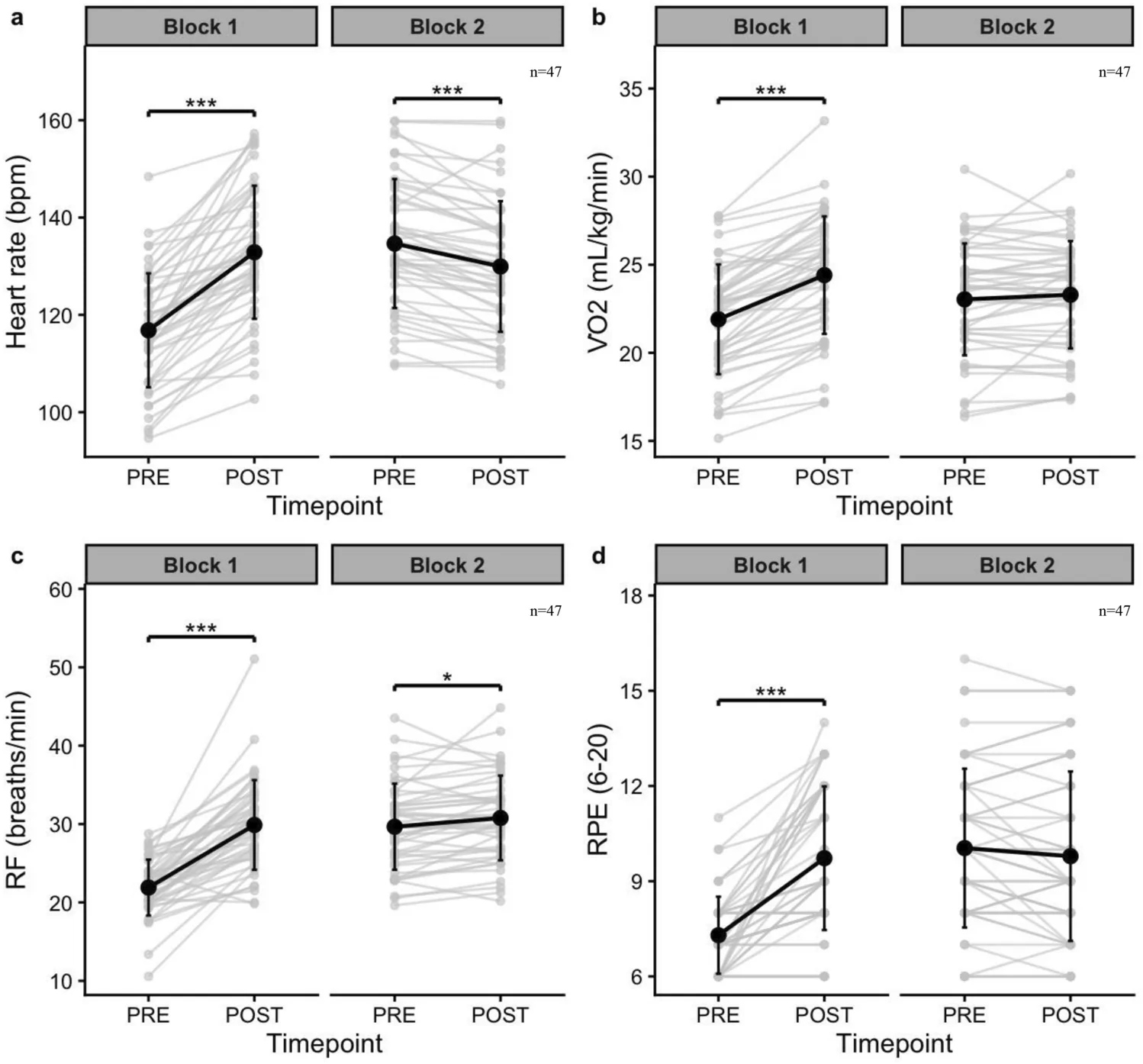
Overall kinetics of **a** heart rate, **b** oxygen consumption (${\dot{\mathrm{V}}\mathrm{O}}_{{2}}$), **c** respiratory frequency (RF) and **d** rating perceived exhaustion (RPE) during submaximal cycling bouts performed before (PRE) and after (POST) the time-to-exhaustion effort in Block1 and Block2. Grey lines represent individual participant responses; black circles denote the group mean and error bars indicate ± 1 SD. Asterisks indicate significant PRE–POST differences within each block (* p < 0.05; *** p < 0.001)

${\dot{\mathrm{V}}\mathrm{O}}_{{2}}$ was significantly higher in highly trained compared to trained participants (β for trained—highly trained =  − 3.35 mL·kg⁻^1^·min⁻^1^, 95% CI − 4.60 to − 2.10, p < 0.001) and higher in male compared to female (β for male–female = 3.46 mL·kg⁻^1^·min⁻^1^, 95% CI 2.18–4.74, p < 0.001). Across groups, ${\dot{\mathrm{V}}\mathrm{O}}_{{2}}$ increased from PRE to POST (β = 1.34 mL·kg⁻^1^·min⁻^1^, 95% CI 1.09–1.60, p < 0.001) but did not differ between Block1 and Block2 (p = 0.958). There was a significant Block*Timepoint interaction (F₁, ₁₂₉ = 62.41, p < 0.001) (see Table 4). Post-hoc tests indicated a marked increase in ${\dot{\mathrm{V}}\mathrm{O}}_{{2}}$ from PRE1 to POST1 (difference = 2.37 mL·kg⁻^1^·min⁻^1^, p < 0.001), whereas the PRE2-POST2 change was smaller and not statistically significant (difference = 0.32 mL·kg⁻^1^·min⁻^1^, p = 0.089). Oxygen consumption at the submaximal workload bouts increased during Block1 (PRE1 to POST1) and remained elevated at the start (PRE2) of Block2, with little further change by the end of Block2 (POST2) (see Fig. 3). The general pattern of an initial increase and subsequent plateau was present in both sexes.

HR was significantly higher in trained than in highly trained participants (β = 11 bpm, 95% CI 5–18, p = 0.001), with no main effect of Sex. Significant main effects were observed for Block (β =  + 7 bpm, 95% CI 6–9, p < 0.001) and Timepoint (β = 5 bpm, 95% CI 4–7, p < 0.001). HR was higher in Block2 than Block1 and higher in general in POST than PRE. A significant Block*Timepoint interaction (F₁, ₁₂₉ = 166.9, p < 0.001; interaction β =  − 19 bpm, 95% CI − 22 to − 16) (see Table 4). Post-hoc tests revealed a significant increase from PRE1 to POST1 as well as a significant decrease from PRE2 to POST2 (see Fig. 3).

RF showed significant main effects of Block (β =  + 4 breaths/min, 95% CI 4–5, p < 0.001) and Timepoint (β =  + 4 breaths/min, 95% CI 4–5, p < 0.001). Values were higher in Block2 and POST compared with Block1 and PRE, respectively, with no differences between performance levels or sexes. The Block*Timepoint interaction was significant (F₁, ₁₂₉ = 64.8, p < 0.001). RF increased strongly from PRE1 to POST1 in Block1 and remained elevated at the beginning of Block2 (PRE2), with further significant increase across the second block (see Fig. 3).

RPE showed to be significantly influenced by the main effects of Block (β =  + 1, 95% CI 1–2, p < 0.001) and Timepoint (β =  + 1, 95% CI 1–2, p < 0.001). No significant main effects or interactions involving Sex or Performance level were shown. A significant Block*Timepoint interaction was present (F₁, ₁₂₉ = 43.68, p < 0.001) (see Table 4). RPE increased markedly from PRE1 to POST1 in Block1 (+ 2, p < 0.001), while the PRE2–POST2 change in Block2 was small and not significant (see Fig. 3).

To compare the explained variance in different variables by the experimental factors, we inspected marginal and conditional R^2^ from the linear mixed models (see Table 4). For DFA α1, the marginal R^2^ was 0.15 and the conditional R^2^ 0.48, thus the fixed effects (Block, Timepoint, Sex, Performance level) indicated 15% of the variability, whereas fixed plus random effects together explained 48% of the variance. For V̇O₂, the marginal and conditional R^2^ were 0.56 and 0.93, respectively; for heart rate, 0.41 and 0.89; for respiration frequency, 0.36 and 0.83; and for RPE, 0.22 and 0.72. Thus, between 22 and 56% of the variance in these variables was explained by the fixed effects alone, and more than 70–90% when individual intercept differences were included.

## Discussion

The aims of the present study were (i) to assess the exercise induced potential alterations of DFA α1 and to compare it to further internal load parameters (HR, ${\dot{\mathrm{V}}\mathrm{O}}_{{2}}$, RPE, RF) assessed during submaximal cycling prior to and following repeated maximal exercise bouts in a larger sample of participants, and (ii) to investigate potential differences between trained and highly trained individuals, as well as between male and female athletes.

The main results showed that:

1. The repeated maximal tests significantly (p < 0.001) induced fatigue, as both V̇O₂peak (53.83 mL·kg⁻^1^·min⁻^1^ ± 10.18, 51.32 mL·kg⁻^1^·min⁻^1^ ± 9.66) and Wmax (360.1 ± 77.8, 336.2 ± 76.0) were significantly lower in RAMP2 compared to RAMP1.
2. Overall, DFA α1 decreased significantly (p < 0.001) from PRE1 (1.32 ± 0.19) to POST1 (1.12 ± 0.34) and remained suppressed in Block2, with no significant differences between POST1, PRE2 (1.14 ± 0.31) and POST2 (1.09 ± 0.35), indicating that DFA α1 tracked both the immediate response to the first maximal effort and the sustained non-recovered state during the subsequent submaximal bouts.
3. ${\dot{\mathrm{V}}\mathrm{O}}_{{2}}$, HR, RF, and RPE increased significantly (p < 0.001) from PRE1 to POST1 and remained elevated at the beginning of the second block (PRE2). From PRE2 to POST2 HR decreased significantly (p < 0.001) and RF increased further (p = 0.045). This reflects an immediate response to the first maximal effort and the sustained non-recovered state during the subsequent submaximal bouts.
4. Performance level showed a significant overall effect (values averaged across all blocks and timepoints during the submaximal bouts) on DFA α1 (p = 0.028), ${\dot{\mathrm{V}}\mathrm{O}}_{{2}}$ (p = 0.004), and HR (p = 0.001) with highly trained participants having higher DFA α1 (1.23, 95% CI [1.14, 1.33])) and V̇O₂ (24.3 mL·kg⁻^1^·min⁻^1^, 95% CI [23.4, 25.1]), and lower HR (123 bpm, 95% CI [119, 128])) compared to trained athletes (DFA α1 1.08, 95% CI [0.98, 1.18], ${\dot{\mathrm{V}}\mathrm{O}}_{{2}}$ 20.9 mL·kg⁻^1^·min⁻^1^, 95% CI [19.9, 21.8]), HR 135 bpm, 95% CI [130, 140]), whereas there was no significant overall effect on RPE or RF.
5. Sex had no significant overall effect on DFA α1, HR, RF and RPE, but a significantly overall effect on ${\dot{\mathrm{V}}\mathrm{O}}_{{2}}$ (p < 0.001), with males exhibiting higher (24.34 mL·kg⁻^1^·min⁻^1^ ± 2.71) values than female participants (20.98 mL·kg⁻^1^·min⁻^1^ ± 3.04).

### DFA α1 as a marker of acute fatigue and non-recovered state during submaximal cycling bouts

Our findings are in line with the emerging literature on DFA α1 assessed during submaximal exercise as a marker of aspects of fatigue (Rogers et al. 2021c; Schaffarczyk et al. 2022a; Van Hooren et al. 2023b). DFA α1 typically decreases with increasing exercise intensity and physiological demands, reflecting a shift from strongly correlated to less correlated values (Gronwald and Hoos 2020). Investigations have shown that DFA α1 remains suppressed during low-intensity exercise after fatiguing tasks, including prolonged running (Rogers et al. 2021c) or high-intensity sessions (Gronwald et al. 2019). Consistent with these reports, we observed a significant decline in DFA α1 during the first block from PRE1 to POST1. At PRE2, DFA α1 remained below the initial pre-fatigue level (PRE1), and there was no significant deviation in POST2. This pattern suggests that the autonomic system did not return to its well-rested state between blocks. This is not unexpected, as both power output and V̇O₂peak were different during the second ramp compared with the first, which plausibly explains the altered DFA α1 response. Importantly, alterations in Wmax and V̇O₂peak indicate that participants showed signs of fatigue, rather than reflecting a simple change in DFA α1.

Previous work has shown both sustained suppression of DFA α1 even after 50 min of heavy exercise (Casties et al. 2006; Gronwald et al. 2019), and rapid normalization during active recovery after interval sessions in well-trained cyclists (Gronwald et al. 2019). Our data extend these findings by demonstrating that within a single session with three maximal efforts, DFA α1 measured during submaximal warm-up (Schaffarczyk et al. 2022a) and successive submaximal bouts shows a non-recovered state.

### Influence of sex and performance level on DFA α1

To the best of our knowledge, this is the first investigation which examines whether sex and performance level modify DFA α1 kinetics during submaximal exercise before and after repeated maximal efforts. In our mixed models, sex did not show a significant main effect or interaction on DFA α1 across the testing protocol. The temporal pattern of DFA α1 decrease and maintained suppression was similar in male and female participants. This aligns with other investigations showing that while male and female differ in some traditional HRV indices (such as linear analysis of low frequency (LF) and high frequency (HF)) (Mendonca et al. 2010) or in overall autonomic function, DFA α1 itself does not display significant sex-specific differences during exercise or in intensity threshold demarcation (Fleitas-Paniagua et al. 2024). Some investigations have reported sex-related contrasts in vagal tone or sympathetic reactivity (Kappus et al. 2015). However, our results show that, at least in the tested population and testing protocol, DFA α1 kinetics under acute and accumulated fatigue does not differ significantly between sexes.

Performance level did show a significant effect on DFA α1. Highly trained participants exhibited higher overall DFA α1 values (1.23 ± 0.28) compared to trained participants (1.09 ± 0.34), consistent with a more favourable autonomic profile and greater fitness (Rogers and Gronwald 2022). This matches earlier findings that higher cardiorespiratory fitness is associated with more robust vagal modulation and HR dynamics during exercise (Tulppo et al. 1998). However, performance level did not substantially alter DFA α1 kinetics as both groups showed significant decreases from PRE1 to POST1 and persistent suppression during Block2. Thus, in this dataset, performance level mainly shifted the absolute level of DFA α1 but did not result in significantly different DFA α1 kinetics across the testing protocol (across PRE1, POST1, PRE2, POST2). This suggests that the DFA α1 kinetics (e.g., acute suppression and sustained lower DFA α1 in the fatigued state) is relatively robust across the herein tested performance levels.

### Influences of the study protocol and grouping factors on DFA α1 and other internal load parameters

An aim of this study was to compare DFA α1 with more established measures of internal load (V̇O₂, HR, RF, RPE) in terms of how strongly they were explained by the effects fitted into the LMM. The higher marginal R^2^ values for V̇O₂, HR, RF, and RPE (see Table 4) indicate that these variables are strongly influenced by the protocol and grouping factors as a large part of their values can be explained from which block and timepoint is considered and who the participant is. In contrast, DFA α1 showed a lower marginal R^2^ (see Table 4), suggesting that a larger proportion of its variability was not directly captured by Block, Timepoint, Sex, or Performance level. A possible explanation for this is that V̇O₂, HR, and RF are closely linked to cardiovascular and metabolic requirements at a given workload (Pendergast 1989), whereas DFA α1 is influenced by the complex interplay of autonomic control, breathing patterns, and other systemic neural and non-neural factors (Tulppo et al. 2005). While Performance level influenced absolute DFA α1 values, relative fatigue-induced changes in DFA α1 were comparable across fitness levels. This finding may help explain why DFA α1-based threshold concepts demonstrate consistent applicability across populations with varying fitness level such as regular and non-regular active females (Schaffarczyk et al. 2023), endurance male athletes (Mateo‐March et al. 2023) or active males (Rogers et al. 2021a). Whereas ${\dot{\mathrm{V}}\mathrm{O}}_{2}$ , HR, and RF closely reflect workload-dependent metabolic demands, DFA α1 behaves as a more variable but potentially more sensitive index of the underlying autonomic state (Gronwald and Hoos 2020) appearing to capture aspects of autonomic regulation that are relatively robust to differences in aerobic fitness, while remaining sensitive to fatigue-related perturbations.

Practically, this implies that due to the high variability, DFA α1 should be interpreted in conjunction with other internal load parameters which are less influenced by e.g. protocol or exercise characteristics, to better interpret physiological response on different bodily systems. For example, a persistent suppression of DFA α1 at a standardised low intensity, together with already elevated HR, and ${\dot{\mathrm{V}}\mathrm{O}}_{2}$, could indicate incomplete recovery or disproportionate autonomic stress, even when performance and perceived exertion appear acceptable (Balagué et al. 2020; Schaffarczyk et al. 2022a; Van Hooren et al. 2023b).

### Strength, limitations and future research

A strength of this study is the integration of a larger number of athletes of different performance level and different sexes, enhancing broader applicability of our findings across athletic populations.

DFA α1 may be sensitive to multiple physiological modulators, including respiratory patterns (Weippert et al. 2015). To mimic real-life scenarios as good as possible, respiration was not standardized in our protocol which could be construed as potential confounding factor but ensures a “free running system” and potential cardiorespiratory coupling mechanisms (Acampa et al. 2021).

In addition, non-linear HRV properties have been shown to vary across the menstrual cycle (Bai et al. 2009) and recent analysis shows no influence of different menstrual cycle phases in naturally-menstruating women during monitoring of submaximal cycling bouts (Schaffarczyk et al. 2025). Although we excluded participants with irregular menstrual cycles, no detailed cycle-related information, including oral contraceptive use, was collected during the laboratory visits, which limits interpretation for female athletes.

Future research should investigate the kinetics of DFA α1 across prolonged exercise durations and a wider spectrum of intensities to determine whether DFA α1 deteriorates from external load parameters such as power output. In the present study, the submaximal exercise phase was performed at fixed absolute power outputs to ensure a clearly low-intensity and standardized physiological condition prior to the ramp tests. It should be noted that the use of these fixed absolute workloads may have resulted in slightly different relative exercise intensities between performance groups, which could have contributed to differences in absolute DFA a1 values. Future studies may examine whether alternative workload prescriptions (e.g., relative intensity or threshold-based approaches) influence DFA α1 responses during such submaximal measurements, particularly when comparing participants of different fitness levels. A further limitation is the absence of a control condition without a second maximal ramp test. Therefore, it cannot be conclusively determined whether the suppressed DFA α1 values observed at POST2 were specifically caused by the second maximal exercise bout or whether DFA α1 would have remained depressed even in the absence of additional maximal exercise. In addition, the present study focused on DFA α1 during a single testing session. Repeating the protocol after full recovery would allow assessment of reproducibility and strengthen conclusions regarding reliability.

## Conclusion

This study shows that DFA α1 measured during submaximal cycling is sensitive to acute fatigue, potentially reflecting a non-recovered organismic state following maximal exercise bouts. DFA α1 decreased after the first maximal cycling bout and remained suppressed throughout the protocol, aligning with sustained elevations in V̇O₂, HR, RF, and RPE. Sex did not influence DFA α1 kinetics, whereas performance level altered absolute values, yet not temporal kinetics.

Compared with conventional markers, DFA α1 displayed a lower marginal coefficient of determination, indicating that a smaller proportion of its variance was explained by the fixed experimental factors (Block, Timepoint, Performance level, Sex). In contrast, a larger share of variability was attributable to between-subject differences and residual variance. This suggests that DFA α1 is less determined by workload and protocol structure and may reflect more individualized autonomic regulatory patterns. Thus, if practitioners aim to use DFA α1 to assess chronic adaptations, repeated measurements should be performed rather than relying on a single assessment. Integrating DFA α1 with traditional physiological measures may enhance the detection of fatigue states in athletes. DFA α1 results need interpretation within the context of individual differences and further work should investigate DFA α1 kinetics across broader exercise conditions and in applied training environments.

## Abbreviations

BMI: Body mass index
DFA α1: Short-term scaling exponent alpha1
HR: Heart rate
HRV: Heart rate variability
RF: Respiratory frequency
RPE: Rating of perceived exertion
$${\dot{\mathrm{V}}\mathrm{O}}_{{2}}$$: Oxygen consumption
${\dot{\mathrm{V}}\mathrm{O}}_{{2}}$peak: Peal oxygen uptake
Wmax: Maximal power output
